# Supplementary figures and images for: Community density patterns estimated by species distribution modeling: The case study of an insect virus interaction
Source: PLoS One. 2025 Jun 10;20(6):e0299183. doi: 10.1371/journal.pone.0299183 (PMC12151466; doi:10.1371/journal.pone.0299183)

A

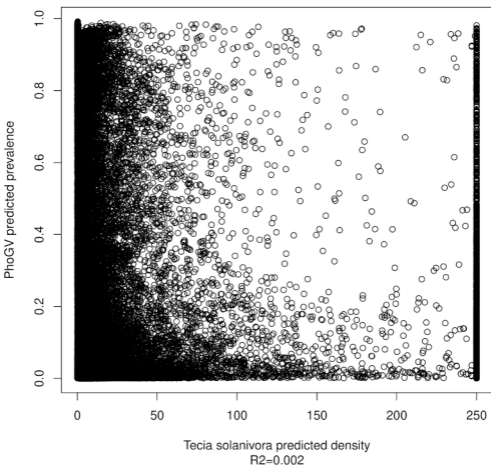

B

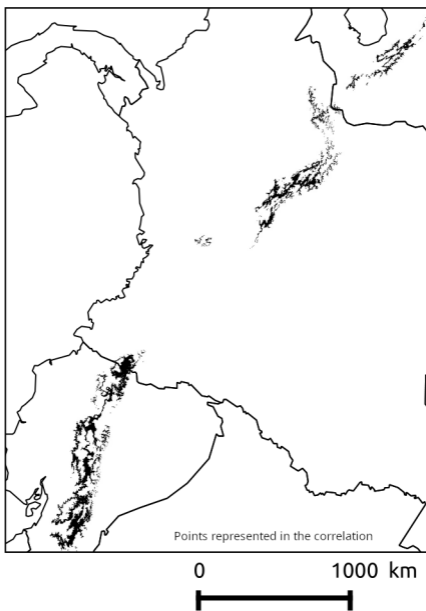

Supplement: S1 Fig — Are presented in this figure only the cells of the map where predictor variables fall in the range where both Tecia solanivora and PhoGV have been sampled. (A) Correlation. (B) Points of the map represented. (PDF) [file pone.0299183.s001.pdf]
