# Supplementary material for: Community density patterns estimated by species distribution modeling: The case study of an insect virus interaction
Source: PLoS One. 2025 Jun 10;20(6):e0299183. doi: 10.1371/journal.pone.0299183 (PMC12151466; doi:10.1371/journal.pone.0299183)
Supplement: S1 Table — (A) Phthorimmaea operculella, (B) Tecia solanivora, and (C) Symetrischemma tangolias. Full model comprised following dependent variables : Alt = Alitude, BIOi = worldclim bioclimatic variables (see details below), their squared values I(.)2, and the belonging to different regions: Central Ecuador (Chimborazo, Bolivar, Tungurahua, Cotopaxi, and Pichincha provinces), Northern Ecuador (Carchi), Central Colombia (Cundinamarca, Boyaca), and Venezuela. BIOi : BIO1 = Annual Mean Temperature, BIO2 = Mean Diurnal Range (Mean of monthly (max temp - min temp)). BIO3 = Isothermality (BIO2/BIO7) (* 100), BIO4 = Temperature Seasonality (standard deviation *100), BIO5 = Max Temperature of Warmest Month, BIO6 = Min Temperature of Coldest Month, BIO7 = Temperature Annual Range (BIO5-BIO6), BIO8 = Mean Temperature of Wettest Quarter, BIO9 = Mean Temperature of Driest Quarter, BIO10 = Mean Temperature of Warmest Quarter, BIO11 = Mean Temperature of Coldest Quarter, BIO12 = Annual Precipitation, BIO13 = Precipitation of Wettest Month, BIO14 = Precipitation of Driest Month, BIO15 = Precipitation Seasonality (Coefficient of Variation), BIO16 = Precipitation of Wettest Quarter, BIO17 = Precipitation of Driest Quarter, BIO18 = Precipitation of Warmest Quarter, BIO19 = Precipitation of Coldest. (DOCX) [file pone.0299183.s003.docx]

| ***Phthorimmaea operculella*** | Estimate | Std. Error | z value | Pr(>\|z\|) |  |
| --- | --- | --- | --- | --- | --- |
| (Intercept) | 1.726e+02 | 1.738e+01 | 9.932 | < 2e-16 | *** |
| Dias | 7.117e-02 | 4.946e-03 | 14.392 | < 2e-16 | *** |
| Northern_Ecuador | 2.968e+00 | 5.825e-01 | 5.095 | 3.49e-07 | *** |
| Central_Colombia | 9.448e+00 | 5.687e-01 | 16.614 | < 2e-16 | *** |
| alt | -1.059e-01 | 9.036e-03 | -11.724 | < 2e-16 | *** |
| bio_1 | -3.080e+00 | 4.826e-01 | -6.382 | 1.74e-10 | *** |
| bio_2 | -3.661e-01 | 6.590e-02 | -5.555 | 2.78e-08 | *** |
| bio_3 | 8.093e-01 | 9.204e-02 | 8.793 | < 2e-16 | *** |
| bio_4 | 3.802e-02 | 3.001e-03 | 12.670 | < 2e-16 | *** |
| bio_5 | -7.415e-01 | 1.561e-01 | -4.750 | 2.03e-06 | *** |
| bio_6 | 2.464e+00 | 2.635e-01 | 9.351 | < 2e-16 | *** |
| bio_11 | 2.393e+00 | 2.674e-01 | 8.948 | < 2e-16 | *** |
| bio_14 | 1.966e-01 | 1.773e-02 | 11.091 | < 2e-16 | *** |
| bio_16 | 3.904e-02 | 4.971e-03 | 7.854 | 4.04e-15 | *** |
| bio_17 | -1.336e-01 | 9.566e-03 | -13.964 | < 2e-16 | *** |
| bio_18 | -1.272e-02 | 3.076e-03 | -4.134 | 3.57e-05 | *** |
| I(alt^2) | 1.536e-05 | 1.461e-06 | 10.514 | < 2e-16 | *** |
| I(bio_1^2) | 1.371e-02 | 2.062e-03 | 6.647 | 2.99e-11 | *** |
| I(bio_4^2) | -1.067e-05 | 2.092e-06 | -5.102 | 3.35e-07 | *** |
| I(bio_5^2) | -2.280e-03 | 5.717e-04 | -3.987 | 6.68e-05 | *** |
| I(bio_6^2) | -5.801e-03 | 1.008e-03 | -5.753 | 8.76e-09 | *** |
| I(bio_7^2) | 9.280e-03 | 9.917e-04 | 9.357 | < 2e-16 | *** |
| I(bio_10^2) | -6.031e-03 | 4.031e-04 | -14.962 | < 2e-16 | *** |
| I(bio_11^2) | -7.552e-03 | 1.131e-03 | -6.679 | 2.41e-11 | *** |
| I(bio_12^2) | -8.615e-06 | 1.207e-06 | -7.140 | 9.36e-13 | *** |
| I(bio_15^2) | -2.400e-03 | 3.084e-04 | -7.781 | 7.18e-15 | *** |
| I(bio_17^2) | 1.750e-04 | 2.346e-05 | 7.462 | 8.50e-14 | *** |
| I(bio_18^2) | 3.443e-05 | 5.722e-06 | 6.017 | 1.78e-09 | *** |
| I(bio_19^2) | 1.439e-05 | 2.850e-06 | 5.050 | 4.42e-07 | *** |

| ***Tecia solaniora*** | Estimate | Std. Error | z value | Pr(>\|z\|) |  |
| --- | --- | --- | --- | --- | --- |
| (Intercept) | 4.931e+01 | 8.635e+00 | 5.710 | 1.13e-08 | *** |
| Dias | 5.959e-02 | 8.335e-03 | 7.150 | 8.68e-13 | *** |
| Northern_Ecuador | 2.360e+00 | 4.160e-01 | 5.673 | 1.40e-08 | *** |
| Central_Colombia | 3.563e+00 | 4.545e-01 | 7.840 | 4.50e-15 | *** |
| Venezuela | 6.332e+00 | 7.538e-01 | 8.400 | < 2e-16 | *** |
| alt | 3.624e-03 | 1.344e-03 | 2.697 | 0.007000 | ** |
| bio_1 | -4.040e+00 | 3.683e-01 | -10.970 | < 2e-16 | *** |
| bio_2 | -1.096e+00 | 1.427e-01 | -7.682 | 1.56e-14 | *** |
| bio_4 | 1.212e-02 | 3.630e-03 | 3.338 | 0.000845 | *** |
| bio_6 | 1.429e+00 | 2.213e-01 | 6.458 | 1.06e-10 | *** |
| bio_10 | 1.824e+00 | 2.744e-01 | 6.649 | 2.96e-11 | *** |
| bio_11 | 1.005e+00 | 1.798e-01 | 5.590 | 2.27e-08 | *** |
| bio_12 | -1.109e-02 | 1.594e-03 | -6.959 | 3.42e-12 | *** |
| bio_14 | 4.264e-01 | 3.559e-02 | 11.983 | < 2e-16 | *** |
| bio_17 | -6.009e-02 | 7.924e-03 | -7.584 | 3.36e-14 | *** |
| bio_18 | -7.990e-03 | 1.668e-03 | -4.790 | 1.67e-06 | *** |
| I(bio_1^2) | 1.611e-02 | 1.393e-03 | 11.566 | < 2e-16 | *** |
| I(bio_2^2) | 3.435e-03 | 5.383e-04 | 6.381 | 1.76e-10 | *** |
| I(bio_3^2) | 3.171e-03 | 5.574e-04 | 5.689 | 1.28e-08 | *** |
| I(bio_4^2) | -2.081e-05 | 3.204e-06 | -6.494 | 8.35e-11 | *** |
| I(bio_5^2) | -2.108e-03 | 4.918e-04 | -4.287 | 1.81e-05 | *** |
| I(bio_6^2) | -1.974e-03 | 2.829e-04 | -6.975 | 3.06e-12 | *** |
| I(bio_7^2) | 5.511e-03 | 8.212e-04 | 6.711 | 1.93e-11 | *** |
| I(bio_9^2) | 4.884e-04 | 5.660e-05 | 8.629 | < 2e-16 | *** |
| I(bio_10^2) | -7.961e-03 | 1.063e-03 | -7.493 | 6.75e-14 | *** |
| I(bio_11^2) | -5.250e-03 | 6.463e-04 | -8.124 | 4.53e-16 | *** |
| I(bio_12^2) | 3.217e-06 | 5.958e-07 | 5.399 | 6.70e-08 | *** |
| I(bio_14^2) | -2.059e-03 | 2.364e-04 | -8.708 | < 2e-16 | *** |
| I(bio_15^2) | 1.380e-03 | 1.349e-04 | 10.230 | < 2e-16 | *** |
| I(bio_18^2) | 1.556e-05 | 2.306e-06 | 6.749 | 1.49e-11 | *** |

| ***Symetrischemma tangolias*** | Estimate | Std. Error | z value | Pr(>\|z\|) |  |
| --- | --- | --- | --- | --- | --- |
| (Intercept) | -1.354e+02 | 1.936e+01 | -6.995 | 2.66e-12 | *** |
| Dias | 2.134e-02 | 3.985e-03 | 5.354 | 8.62e-08 | *** |
| Northern_Ecuador | -3.295e+00 | 3.271e-01 | -10.074 | < 2e-16 | *** |
| alt | 9.817e-02 | 1.040e-02 | 9.440 | < 2e-16 | *** |
| bio_1 | -2.812e+00 | 3.105e-01 | -9.057 | < 2e-16 | *** |
| bio_2 | 1.402e+00 | 2.429e-01 | 5.773 | 7.80e-09 | *** |
| bio_3 | -2.233e-01 | 5.012e-02 | -4.456 | 8.36e-06 | *** |
| bio_4 | 4.183e-02 | 4.280e-03 | 9.772 | < 2e-16 | *** |
| bio_6 | -2.272e-01 | 3.718e-02 | -6.111 | 9.93e-10 | *** |
| bio_8 | -1.322e-01 | 1.678e-02 | -7.880 | 3.27e-15 | *** |
| bio_11 | 2.144e+00 | 2.421e-01 | 8.853 | < 2e-16 | *** |
| bio_12 | 4.413e-02 | 3.541e-03 | 12.462 | < 2e-16 | *** |
| bio_13 | 1.324e-01 | 1.588e-02 | 8.338 | < 2e-16 | *** |
| bio_14 | 3.492e-01 | 3.920e-02 | 8.908 | < 2e-16 | *** |
| bio_15 | -1.077e-01 | 2.754e-02 | -3.911 | 9.21e-05 | *** |
| bio_16 | -9.066e-02 | 8.877e-03 | -10.213 | < 2e-16 | *** |
| bio_17 | -2.946e-01 | 2.498e-02 | -11.796 | < 2e-16 | *** |
| bio_18 | -1.270e-02 | 2.559e-03 | -4.963 | 6.93e-07 | *** |
| bio_19 | 4.902e-02 | 8.231e-03 | 5.955 | 2.60e-09 | *** |
| I(alt^2) | -1.678e-05 | 1.680e-06 | -9.988 | < 2e-16 | *** |
| I(bio_1^2) | 1.475e-02 | 1.467e-03 | 10.059 | < 2e-16 | *** |
| I(bio_2^2) | -7.388e-03 | 1.120e-03 | -6.599 | 4.14e-11 | *** |
| I(bio_4^2) | -3.489e-05 | 3.997e-06 | -8.730 | < 2e-16 | *** |
| I(bio_10^2) | -4.436e-03 | 3.930e-04 | -11.285 | < 2e-16 | *** |
| I(bio_11^2) | -6.128e-03 | 8.519e-04 | -7.193 | 6.32e-13 | *** |
| I(bio_12^2) | -1.980e-05 | 1.804e-06 | -10.975 | < 2e-16 | *** |
| I(bio_14^2) | -3.489e-03 | 3.998e-04 | -8.728 | < 2e-16 | *** |
| I(bio_15^2) | -1.786e-03 | 2.876e-04 | -6.210 | 5.29e-10 | *** |
| I(bio_16^2) | 7.372e-05 | 7.763e-06 | 9.497 | < 2e-16 | *** |
| I(bio_17^2) | 7.297e-04 | 6.080e-05 | 12.001 | < 2e-16 | *** |
| I(bio_18^2) | 1.900e-05 | 4.243e-06 | 4.477 | 7.59e-06 | *** |
| I(bio_19^2) | -1.116e-04 | 1.722e-05 | -6.484 | 8.91e-11 | *** |
