# Supplementary material for: Community density patterns estimated by species distribution modeling: The case study of an insect virus interaction
Source: PLoS One. 2025 Jun 10;20(6):e0299183. doi: 10.1371/journal.pone.0299183 (PMC12151466; doi:10.1371/journal.pone.0299183)
Supplement: S2 Table — Alt = Alitude, BIO9 = Mean temperature of the driest quarter, BIO12 = Annual precipitation. . Alt = Alitude, BIO9 = Mean Temperature of Driest Quarter, BIO12 = Annual Precipitation. (DOCX) [file pone.0299183.s004.docx]

Supplementary table 2.

Results of stepwise polynomial generalized linear model regression for pohGV prevalence in *Tecia solanivora*. Alt = Alitude, BIO9 = Mean Temperature of Driest Quarter, BIO12 = Annual Precipitation*.* % Model Deviance

|  | Estimate | Std._Error | z_value | % Model Deviance | Pr(>\|z\|) |
| --- | --- | --- | --- | --- | --- |
| (Intercept) | 91.03 | 20.5 | 4.44 |  | 9.15e-06 |
| alt | -0.015 | 0.004 | -3.54 | 20.1 | 4.05e-04 |
| bio9 | -0.328 | 0.068 | -4.84 | 8.6 | 1.32e-06 |
| bio12 | -0.014 | 0.002 | -8.54 | 71.3 | 1.32e-17 |
