# Supplementary material for: Community density patterns estimated by species distribution modeling: The case study of an insect virus interaction
Source: PLoS One. 2025 Jun 10;20(6):e0299183. doi: 10.1371/journal.pone.0299183 (PMC12151466; doi:10.1371/journal.pone.0299183)
Supplement: Attachment — Submitted filename: rebutal letter.odt [file pone.0299183.s005.odt]

PONE-D-24-05136
Community density patterns estimated by species distribution modeling: the case study of an insect virus interaction
PLOS ONE

Dear Dr. Dupas,

Thank you for submitting your manuscript to PLOS ONE. After careful consideration, we feel that it has merit but does not fully meet PLOS ONE’s publication criteria as it currently stands. Therefore, we invite you to submit a revised version of the manuscript that addresses the points raised during the review process.

Please submit your revised manuscript by Jun 20 2024 11:59PM. If you will need more time than this to complete your revisions, please reply to this message or contact the journal office at plosone@plos.org. When you're ready to submit your revision, log on to https://www.editorialmanager.com/pone/ and select the 'Submissions Needing Revision' folder to locate your manuscript file.

Please include the following items when submitting your revised manuscript:
A rebuttal letter that responds to each point raised by the academic editor and reviewer(s). You should upload this letter as a separate file labeled 'Response to Reviewers'.
A marked-up copy of your manuscript that highlights changes made to the original version. You should upload this as a separate file labeled 'Revised Manuscript with Track Changes'.
An unmarked version of your revised paper without tracked changes. You should upload this as a separate file labeled 'Manuscript'.
If you would like to make changes to your financial disclosure, please include your updated statement in your cover letter. Guidelines for resubmitting your figure files are available below the reviewer comments at the end of this letter.
We included our updated statements in our cover letter

If applicable, we recommend that you deposit your laboratory protocols in protocols.io to enhance the reproducibility of your results. Protocols.io assigns your protocol its own identifier (DOI) so that it can be cited independently in the future. For instructions see: https://journals.plos.org/plosone/s/submission-guidelines#loc-laboratory-protocols. Additionally, PLOS ONE offers an option for publishing peer-reviewed Lab Protocol articles, which describe protocols hosted on protocols.io. Read more information on sharing protocols at https://plos.org/protocols?utm_medium=editorial-email&utm_source=authorletters&utm_campaign=protocols.

We look forward to receiving your revised manuscript.
Kind regards,
Clement Ameh Yaro, Ph.D
Academic Editor
PLOS ONE
Journal Requirements:
When submitting your revision, we need you to address these additional requirements.
1. Please ensure that your manuscript meets PLOS ONE's style requirements, including those for file naming. The PLOS ONE style templates can be found at 
https://journals.plos.org/plosone/s/file?id=wjVg/PLOSOne_formatting_sample_main_body.pdf and 
https://journals.plos.org/plosone/s/file?id=ba62/PLOSOne_formatting_sample_title_authors_affiliations.pdf

We ensured file naming and style templates in this revision

2. Please update your submission to use the PLOS LaTeX template. The template and more information on our requirements for LaTeX submissions can be found at http://journals.plos.org/plosone/s/latex.

We reworked the manustript using a PLOS latex template

3. In your Methods section, please provide additional information regarding the permits you obtained for the work. Please ensure you have included the full name of the authority that approved the field site access and, if no permits were required, a brief statement explaining why.

We added this information

4. We note that the grant information you provided in the ‘Funding Information’ and ‘Financial Disclosure’ sections do not match. 
When you resubmit, please ensure that you provide the correct grant numbers for the awards you received for your study in the ‘Funding Information’ section.
Done
5. Thank you for stating the following financial disclosure: 
"Programme ECOFOR Ecosystèmes Tropicaux, Projet ENTOAND 2007-2011"
Please state what role the funders took in the study.  If the funders had no role, please state: ""The funders had no role in study design, data collection and analysis, decision to publish, or preparation of the manuscript."" 
If this statement is not correct you must amend it as needed. 
Please include this amended Role of Funder statement in your cover letter; we will change the online submission form on your behalf.
Ok The funders had no role in study design...
6. Thank you for stating the following in the Acknowledgments Section of your manuscript: 
"This work has been founded by ECOFOR program ENTOAND 2007-2011"
We note that you have provided funding information that is not currently declared in your Funding Statement. However, funding information should not appear in the Acknowledgments section or other areas of your manuscript. We will only publish funding information present in the Funding Statement section of the online submission form. 
Please remove any funding-related text from the manuscript and let us know how you would like to update your Funding Statement. Currently, your Funding Statement reads as follows: 
"Programme ECOFOR Ecosystèmes Tropicaux, Projet ENTOAND 2007-2011"
Please include your amended statements within your cover letter; we will change the online submission form on your behalf.
Ok

[Note: HTML markup is below. Please do not edit.]

Reviewers' comments:

Reviewer's Responses to Questions
Comments to the Author

1. Is the manuscript technically sound, and do the data support the conclusions?

The manuscript must describe a technically sound piece of scientific research with data that supports the conclusions. Experiments must have been conducted rigorously, with appropriate controls, replication, and sample sizes. The conclusions must be drawn appropriately based on the data presented. 
Reviewer #1: Partly
Reviewer #2: Yes

2. Has the statistical analysis been performed appropriately and rigorously? 
Reviewer #1: I Don't Know
Reviewer #2: Yes

3. Have the authors made all data underlying the findings in their manuscript fully available?

The PLOS Data policy requires authors to make all data underlying the findings described in their manuscript fully available without restriction, with rare exception (please refer to the Data Availability Statement in the manuscript PDF file). The data should be provided as part of the manuscript or its supporting information, or deposited to a public repository. For example, in addition to summary statistics, the data points behind means, medians and variance measures should be available. If there are restrictions on publicly sharing data—e.g. participant privacy or use of data from a third party—those must be specified.
Reviewer #1: Yes
Reviewer #2: Yes

4. Is the manuscript presented in an intelligible fashion and written in standard English?

PLOS ONE does not copyedit accepted manuscripts, so the language in submitted articles must be clear, correct, and unambiguous. Any typographical or grammatical errors should be corrected at revision, so please note any specific errors here.
Reviewer #1: Yes
Reviewer #2: Yes

5. Review Comments to the Author

Please use the space provided to explain your answers to the questions above. You may also include additional comments for the author, including concerns about dual publication, research ethics, or publication ethics. (Please upload your review as an attachment if it exceeds 20,000 characters)
Reviewer #1: This is an interesting study demonstrating a methodological framework to study patterns of pathogen prevalence and host density. However, it requires much additional work before being acceptable for publication.

My main objection lies in the apparent “hybrid” nature of the study. Whereas the introduction is mostly about developing a new methodological approach applied to a particular case, the rest of the paper concerns the specifics of the system under study.

I would challenge the purportedly methodological nature of the paper. Developing a new approach would require (i) to test the method with simulated data to establish its efficiency and (ii) to compare its results with alternative methods to assess is performance. These steps have not been undertaken in the study.

My suggestion is that the authors focus in the analysis of their specific virus-insect system and indicate somewhere in the discussion that their approach might be valuable given the difficult applicability of other approaches.

We revisited the paper in order to focuss on the analysis of our specific system. The manuscrit was introduced on the question of the lack of data regarding the natural impact of viruses on insect pests despite their use in biological control. All the manustript was carefully reviewed for the english writing with machine learning. 


Specific comments
Abstract 5.- It is not clearly shown what this results means biologically.

51. insert “on” between “relies” and “long”.
ok

53-54. “Casuality is still not inferred…” It is not entirely clear what is meant here.
In the present version, the work was no longer introduced from the fundamental question of characterizing species interactions from field or experimental data, but from the more applied question of the natural impact of insect viruses on agricultural pests. In this new context, the question of causality versus correlation has been removed. 

196. “precipitation being the most significant…” This can be misleading because p-values do not indicate of the size of the effect. A very low p-value suggests a clear difference but not necessarily a large one. So being “the most significant” is not particularly informative in terms of biology.
It is true. We analysed percentage of deviance explained and changed to “precipitation explained the most residual deviance (71.3%)”

I could not find any captions for figures 1 and 2. Apparently that of S1 Fig. corresponds actually to Fig. 1, but then what is the S1 figure included in supplementary information? Likewise, S2_Fig.pdf is not referred to in the text and the S1 R script is identified as S2 in line 285.

Thank you. The fig. 1 and 2 captions were included in the text, and S1 and S2 Fig captions at the end of the manuscrit as requested in Plos One formating guidelines. 
Reviewer #2: From my point of view, the document is well structured and supported by the data presented. This can be considered an interesting contribution to a very little studied subject in the neotropical region and particularly in countries such as Ecuador.

6. PLOS authors have the option to publish the peer review history of their article (what does this mean?). If published, this will include your full peer review and any attached files.


If you choose “no”, your identity will remain anonymous but your review may still be made public.


Do you want your identity to be public for this peer review? For information about this choice, including consent withdrawal, please see our Privacy Policy.
Reviewer #1: No
Reviewer #2: No
